# Identification of Novel Placentally Expressed Aspartic Proteinase in Humans

**DOI:** 10.3390/ijms18061227

**Published:** 2017-06-08

**Authors:** Marta Majewska, Aleksandra Lipka, Grzegorz Panasiewicz, Marek Gowkielewicz, Marcin Jozwik, Mariusz Krzysztof Majewski, Bozena Szafranska

**Affiliations:** 1 Department of Human Physiology, Faculty of Medical Sciences, University of Warmia and Mazury in Olsztyn, Warszawska Str 30, 10-082 Olsztyn, Poland; mariusz.majewski@uwm.edu.pl; 2 Department of Gynecology and Obstetrics, Faculty of Medical Sciences, University of Warmia and Mazury in Olsztyn, Niepodleglosci Str 44, 10-045 Olsztyn, Poland; aleksandra.lipka@uwm.edu.pl (A.L.); marekgowkielewicz@gmail.com (M.G.); jozwik@interia.eu (M.J.); 3 Department of Animal Physiology, Faculty of Biology and Biotechnology, University of Warmia and Mazury in Olsztyn, Oczapowskiego Str 1A, 10-719 Olsztyn, Poland; panasg@uwm.edu.pl (G.P.); szafran@uwm.edu.pl (B.S.)

**Keywords:** cDNA, gDNA, human PAG-L, placenta, trophectoderm

## Abstract

This study presents pioneering data concerning the human pregnancy-associated glycoprotein-Like family, identified in the genome, of the term placental transcriptome and proteome. RNA-seq allowed the identification of 1364 bp *hPAG-L/pep* cDNA with at least 56.5% homology with other aspartic proteinases (APs). In silico analyses revealed 388 amino acids (aa) of full-length hPAG-L polypeptide precursor, with 15 aa-signal peptide, 47 aa-blocking peptide and 326 aa-mature protein, and two Asp residues (D), specific for a catalytic cleft of the APs (VVFDTGSSNLWV91-102 and AIVDTGTSLLTG274-285). Capillary sequencing identified 9330 bp of the *hPAG-L* gene (Gen Bank Acc. No. KX533473), composed of nine exons and eight introns. Heterologous Western blotting revealed the presence of one dominant 60 kDa isoform of the hPAG-L amongst cellular placental proteins. Detection with anti-pPAG-P and anti-Rec pPAG2 polyclonals allowed identification of the hPAG-L proteins located within regions of chorionic villi, especially within the syncytiotrophoblast of term singleton placentas. Our novel data extend the present knowledge about the human genome, as well as placental transcriptome and proteome during term pregnancy. Presumably, this may contribute to establishing a new diagnostic tool for examination of some disturbances during human pregnancy, as well as growing interest from both scientific and clinical perspectives.

## 1. Introduction

Pregnancy-associated glycoproteins (PAGs) belong to a superfamily of aspartic proteinases (AP), which also include mammalian pepsins (A, C and F), cathepsins (D and E), renin and numerous other enzymes such as parasite plasmepsins and retroviral enzymes [1,2]. All AP members possess a two-bilobe structure with a cleft capable of short peptide binding and are classified into two subfamilies: catalytically active or potentially inactive due to several amino acid (aa) substitutions within two domains creating the binding cleft [3,4]. Among APs, pepsins fulfil digestive functions outside the cells, whereas cathepsin D and E are typical intracellular enzymes generally localized in the lysosomal compartment that provides the acidic environment necessary to accomplish their catalytic functions [5,6]. On the other hand, PAG-Like (PAG-L) family products revealed properties as various chorionic signaling ligands interacting with gonadal and extra-gonadal gonadotropin receptors of early pregnant pigs [7], as well as cyclic pigs and cows [8].

In humans, various AP members are involved in the development of a variety of diseases, e.g., hypertension, gastric ulcers, acquired immunodeficiency syndrome, malaria, lysosomal muscular dystrophy and neoplastic diseases, etc. [2,9]. APs are also involved in defense against infections, tumor cells, cancer and in the development of atopic dermatitis [10,11,12].

During gestation, APs may also play important roles during implantation and in the establishment of early pregnancy, since chorionic expression of cathepsins B and L differ in normal and abnormal deciduas [13], whereas an imbalance of the cathepsin–cystatin system causes miscarriages [14]. Decreased activity of cathepsin E might also be responsible for induction of miscarriages by decreased decidual expression, especially in macrophages of patients with recurrent pregnancy loss [15].

Decreased PAG family expression also occurs during gestation disorders. The PAG family originates from a progene duplication or its fragments and positive selection of these genes [16]. To date, the entire exon-intron structures of only four *PAG* genes have been identified within some genomes, bovine—*bPAG1* [17], *bPAG2* [18], porcine—*pPAG2* [19] and beaver—*CfPAG-L* [20]. The *PAGs* are characterized by a conserved structure that includes nine exons and eight introns [1,2]. All mammalian *PAGs* and related *PAG-L* genes are the most closely homologous to the pepsins [18,21].

Mammalian placenta is a unique organ essential for fetal growth, development and survival in the uterus [22], with complex of biomolecular interactions between the fetus and mother that provide structural and biochemical barriers between both compartments [23]. The human placenta is hemochorial (maternal blood is in direct contact with fetal trophoblast) and discoidal in shape with villous materno-fetal interdigitations [24]. Within each placenta type developed in various eutherians, trophoblast forms the outer layer of a blastocyst, then expands into the trophectoderm—chorionic epithelium, which together with the endometrium, forms the placenta [25]. Within a very precise feto-maternal interface [26,27], specific expression of the PAGs is cell- and pregnancy stage-dependent [1,2].

Many purified native or several recombinant proteins, required for anti-PAG sera production, have led to the establishment of various pregnancy diagnoses, based on PAG-L detection in maternal blood or milk by radioimmunological (RIA) and immunoenzymatic (ELISA) tests [1]. These PAG tests are useful for detecting abnormalities during pregnancy in cattle [28,29] and to predict miscarriages after embryo transfers [30]. Since the varying PAG concentration depends on the number of healthy embryos/fetuses, it is higher in females with twin than single pregnancies and can also differ due to the fetal sex and breed in many domestic and some wild ruminants [1,31].

In view of both the commitment of these chorionic proteins in the course of pregnancy and evolutionary persistence of the *PAG* genes in various eutherian species [1,2], there is growing interest in examining whether they are also present in humans. The subsistence of this unique PAG/PAG-L family has not yet been studied in humans.

The objective of this study was to identify the existence of the PAG-L family in humans: (1) placental transcriptome; (2) genome; (3) placental proteome, including immuno-detection of protein profiles and cellular localization in the term placenta.

## 2. Results

### 2.1. Identification of cDNA Sequence Originating from Term Placental Transcriptome

The *hPAG-L* sequence was identified by two methods. The performed RNA-seq generated a total of 71,271,470 pairs of raw reads and 58,547,248 trimmed pair reads (82%) obtained after removing TruSeq adaptors and low quality reads. TRINITY software enabled de novo assembly of 102,357 contigs. The reconstructed contigs were analyzed for similarity to the AP superfamily, which allowed identification of a 1364 bp cDNA sequence of the placental *hPAG-L* transcript.

**Figure 1:**
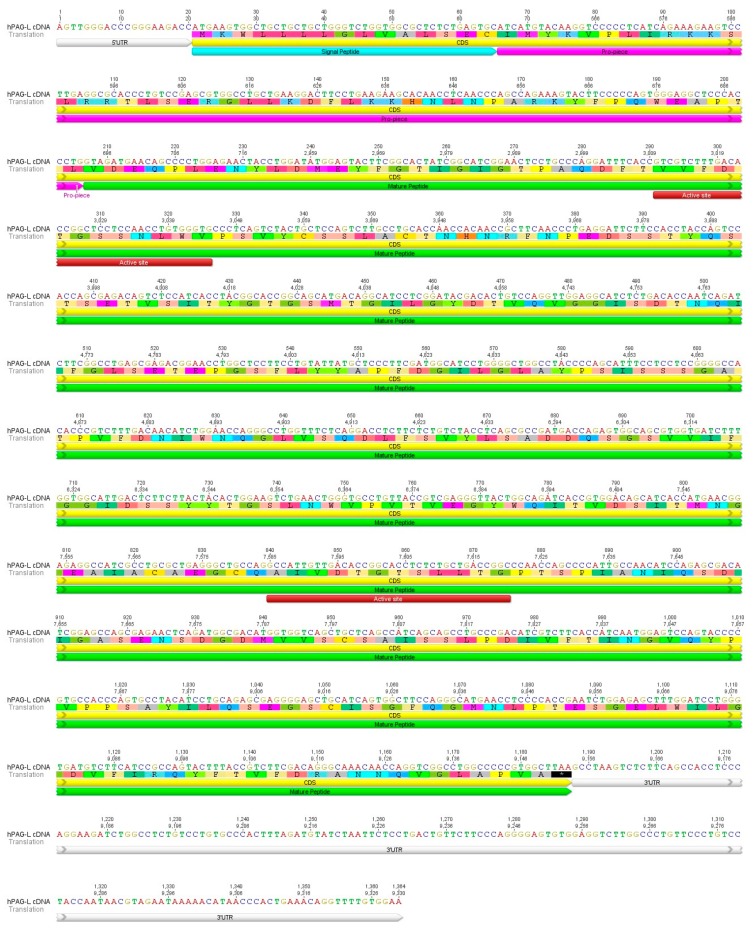
Identified cDNA sequence of 1364 bp human *Pregnancy-Associated Glycoprotein-like* (*hPAG-L*) encoding full-length 388 amino acids (aa) of polypeptide precursor. 5′- and 3′-untranslated regions (5′UTR and 3′UTR), 15 aa-signal peptide, 47 aa-blocking peptide, 326 aa-mature protein, and active sites creating catalytic clefts are indicated.

In addition, cDNA evaluation by capillary sequencing firmly confirmed the nucleotide sequence originating from the RNA-seq. Five pairs of homological *PAG-L* primers applied for PCRs allowed obtaining the entire cDNA sequence (1364 bp), named *hPAG-L*, and deposited in GenBank database (Acc. No. KX856064). Among the 109 electrophoresed, gel-out purified and sequenced cDNA amplicons, 80 high quality chromatograms (HQ range: 50–93.9%) were applied to estimate the coding (CDS) and non-coding untranslated regions (UTR). Among the identified 1364 bp of cDNA sequence, 1167 bp were determined as CDS, 20 bp as 5′UTR and 177 bp as 3′UTR (Figure 1).

A megablast of the *hPAG-L* cDNA revealed 85–99% homology with mammalian pepsinogens A, (peps) whereas Query Cover (QC) ranged from 68% to 100% in various species (e.g., *Homo sapiens*, *Gorilla gorilla*, *Pan troglodytes*, *Pongo abeli*, *Nomascus leucogenys*, *Macaca Fascicularis*, *Colobus angolensis*, *Rhinopithecus roxellana*, *Camelus ferus*, *Ursus maritimus*, *Mustela putorius*, *Canis lupus*, *Leptonychotes weddellii*).

Pair-wise alignment (Geneious^®^ 8.1.7) of the *hPAG-L* cDNA sequence with various AP members indicated the highest identity with: human *pep A* (99%; NM_001079808.3), zebra *PAG* (66.2%; AF036952); mouse *pep F* (65.3%; AF240776.1); equine *PAG* (*ePAG*; 65.9%; L38511); human *pep C* (64.2%; J04443.1); with *pPAG2* (64%; L34361.1); beaver *PAG-L* (*CfPAG-L*; 63.1%; KU245742.1); and also human *cathepsin E* (61.4%; NM_001910.3); *cathepsin D* (59.1%; NM_001909.4); *napsin A* (56.5%; NM_004851.2) and *renin* (56.5%; NM_000537.3). Due to the highest identity of *hPAG-L* with human pepsinogens (*PGPGA4*, *PGA3* and *PGA5*), the identified placental AP can be also named as *hPAG-L/pep*.

The *hPAG-L/pep* cDNA allowed identification of a 388 aa-polypeptide precursor (Geneious^®^ 8.1.7). The entire placental AP precursor revealed the highest aa identity with: human pep A (99.2%); mouse pep F (56.2%); pPAG2 (51.2%); cathepsin E (53%); pep C (50%); cathepsin D (44%); napsin A (40.5%) and renin (36.3%).

### 2.2. Identification of cDNA Sequence Originating from Term Placental Transcriptome

The hPAG-L/pep polypeptide precursor retains two highly conserved domains (NH_2_ and COOH), specific to other members of AP superfamily. Geneious^®^ 8.1.7 allowed identification of 15 aa-signal peptide (SP), 47 aa-blocking peptide and 326 aa-mature hPAG-L/pep precursor (Figure 1).

The identified SP aa sequence of the hPAG-L/pep shared the highest similarity with SP of the human pep A and it varied with the other members of the AP family in different species (Table 1).

Multiple alignments of the various APs enabled the prediction of 47 aa-blocking peptide (16–62 aa) of the hPAG-L/pep precursor that shared the highest homology with human pep A, whereas identity is equal/similar with peps C and F as well as other PAGs (Table 2).

A putative cleavage position was predicted between PTL60–62 of the blocking pro-piece and VDE63–65 of the mature hPAG-L/pep precursor (Figure 1). Two Asp residues (D), specific for the catalytic cleft of AP were predicted at positions 94 aa in the NH_2_-terminus (VVFDTGSSNLWV91–102) and 277 aa in the COOH-terminus (AIVDTGTSLLTG274–285 Figure 1) of the hPAG-L precursor. The sequences of the NH_2_- and COOH-terminal domain of the hPAG-L/pep are identical to human pep A and very homologous to many other APs (Table 3). Surprisingly, no potential *N*-glycosylation site was predicted in the hPAG-L/pep precursor. In addition, in silico analyses permitted the identification of the molecular mass of the hPAG-L/pep polypeptide precursor (41.993 kDa) and its electrostatic property (pI 3.93).

### 2.3. Identification of the hPAG-L/pep Sequence within the Human Genome

**Figure 2:**
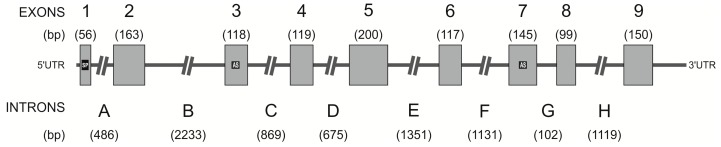
Structural organization of the *hPAG-L/pep* gene. Exons (1–9) are boxed with their sizes shown in parenthesis above each box. The introns (A–H) are represented as lines with their sizes shown below each. Exons 3 and 7, which contain the catalytic aspartic acids at the active site, are shaded. The untranslated regions are represented by lines labeled 5′UTR and 3′UTR. Abbreviations: SP—signal peptide; AS—active site sequences coding domains 1 and 2 of the catalytic cleft.

Homological primers (19 pairs) amplified six human gDNA templates, producing multiple *hPAG-L/pep* amplicons. Approximately 300 *hPAG-L/pep* gDNA sequenced amplicons were obtained. Only 197 chromatograms revealed high quality (HQ range: 40–98.2%) that were analyzed with Geneious^®^ 8.1.7. A thorough analysis led to the identification of a 9330-bp genomic sequence of the entire *hPAG-L/pep* and deposition in the GenBank database (Acc. No. KX533473). The novel *hPAG-L/pep* gDNA sequence is composed of nine exons (56–200 bp) and eight introns (A–H; 102–2233 bp; Figure 2).

The lengths of the *hPAG-L/pep* exons were generally very similar, or even the same, as in other *PAGs* (*bPAG1*: Acc. No. AH003454.1, *bPAG2*: Acc. No. NM_176614.1, *pPAG2*: Acc. Nos.: U39198–9; U41421–4; U39762–3; KF471015.1; KF492695.1; KF500427.1; KF527576.1; KF537535.1 and *CfPAG-L*: Acc. No. KX377932). However, the intron lengths varied, except for E and G introns, which were comparable (Table 4).

Two coded D residues within both domains creating a catalytic cleft (a feature of the AP members) were localized within exons 3 and 7 of the *hPAG-L/pep* (Figure 2). All exon-intron junctions with 5′ donor and 3′ acceptor sites were identified (Table 5). The sequences between each of the intron-exon junctions were determined and conformed to the standard gt-ag rule for 5′ donor and 3′ acceptor sites. The de novo identified *hPAG-L* gene is composed of: 24.7% A; 27.4% C; 25.6% G; 22.3% T and 53.0% G+C (Geneious^®^ 8.1.7.).

Megablast alignments of the *hPAG-L/pep* gDNA revealed the highest homology with human sequences with various identity (ID) and QC: pepsin-L (e.g., *pep 3*, *4* and *pepsin A*; ID 98–100%, 3–15% QC), chromosome 11 BAC and FOSMID clones (99%), pepsin/pep A–L sequences in different species: e.g., *Pan troglodytes* (98% ID, 20% QC), *Macaca fuscata* (92% ID, 26% QC) and *Papio anubis* (92% ID, 18% QC). Furthermore, pairwise alignments (Geneious^®^ 8.1.7) of the *hPAG-L/pep* indicated higher homology of each exon (52.1–78.6%) and lower intronic homology (25.4–58.5%) with other *PAGs* identified previously in different species (Table 6).

### 2.4. Identification of Placental hPAG-L/pep Protein

**Figure 3:**
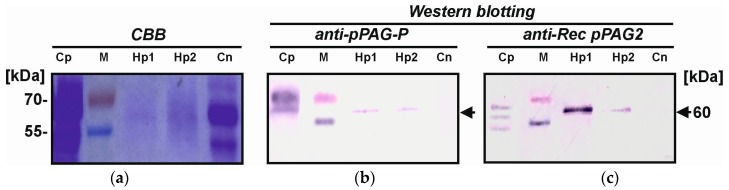
Identification of cellular human hPAG-L term placental proteins (10 μg/sample) separated by (**a**) SDS-PAGE and stained by Coomassie Brilliant Blue (CBB); Heterologous Western blottings with (**b**) anti-porcine PAG-P polyclonals (1:300); (**c**) Western analysis with anti-Rec pPAG2 (1:50). Abbreviations: Cp—positive control (porcine secretory chorionic proteins; 77 dpc); M—molecular marker; Hp1 and Hp2—human placental proteins; Cn—negative control (secretory endometrial proteins; 10 day of cycle). Arrow indicates a dominant hPAG-L isoform.

Isolated cellular placental proteins (2.61–5.15 µg/µL) allowed identification of hPAG-L/pep profiles specific for the term gestation. Heterologous Western blotting with either anti-pPAG-P or anti-Rec pPAG2 polyclonals revealed only one dominant 60 kDa hPAG-L protein, similar in molecular mass to a positive control of multiple porcine placental proteins originated from in vitro culture of 77 dpc-chorionic explants (Figure 3). A lack of any signals in the negative control (secretory endometrial proteins; 10 day of cycle) confirmed the correctness of the immunoblotting.

### 2.5. Identification of Cellular hPAG-L/pep Localization

**Figure 4:**
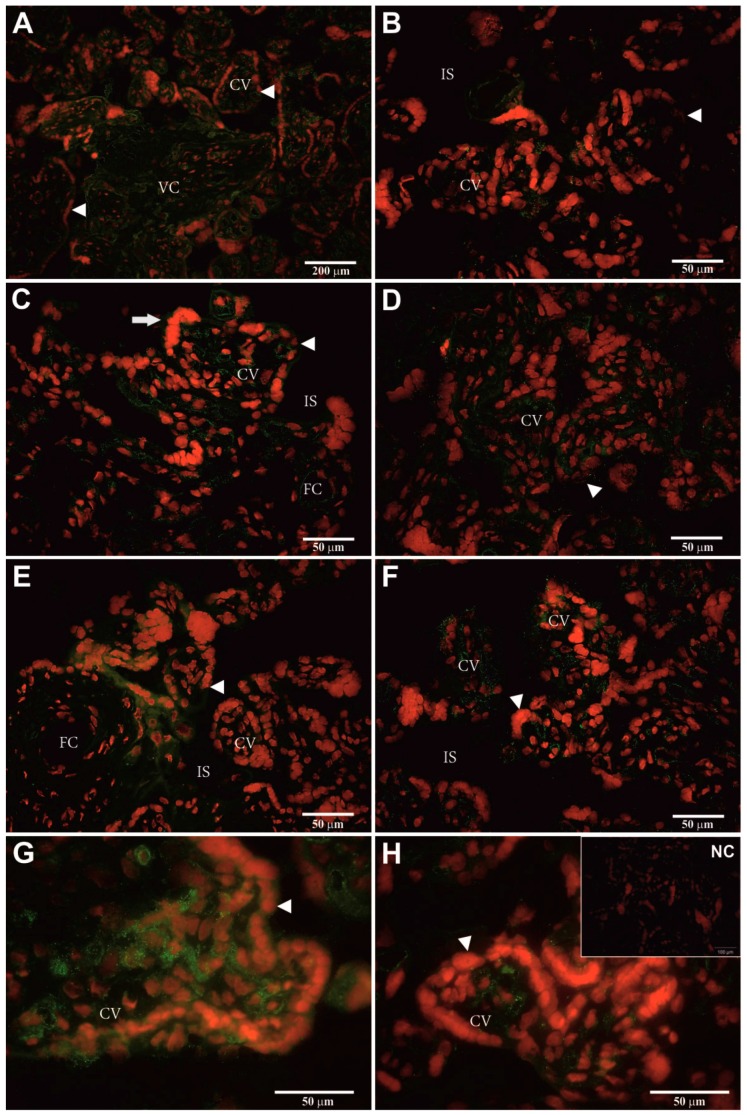
Heterologous immuno-localization of the hPAG-L/pep proteins within sections of a human term placenta identified with polyvalent anti-porcine PAG (anti-pPAG-P) polyclonals (**A**–**H**), visualized by goat anti-rabbit IgG-conjugated with Alexa 488 fluorophore (green) among all nuclei stained by propidium iodide (red). Human placenta section—used as negative control (NC; insert in H) with omitted polyvalent anti-pPAG-P polyclonals. The size bars are 50 µm (**B**–**H**), 100 µm (NC) and 200 µm (**A**). Abbreviations: arrowheads—syncytiotrophoblast; arrows—clusters of the syncytiotrophoblast nuclei; CV—chorionic villi; VC—villous core; IS—intervillous space; FC—fetal capillary.

**Figure 5:**
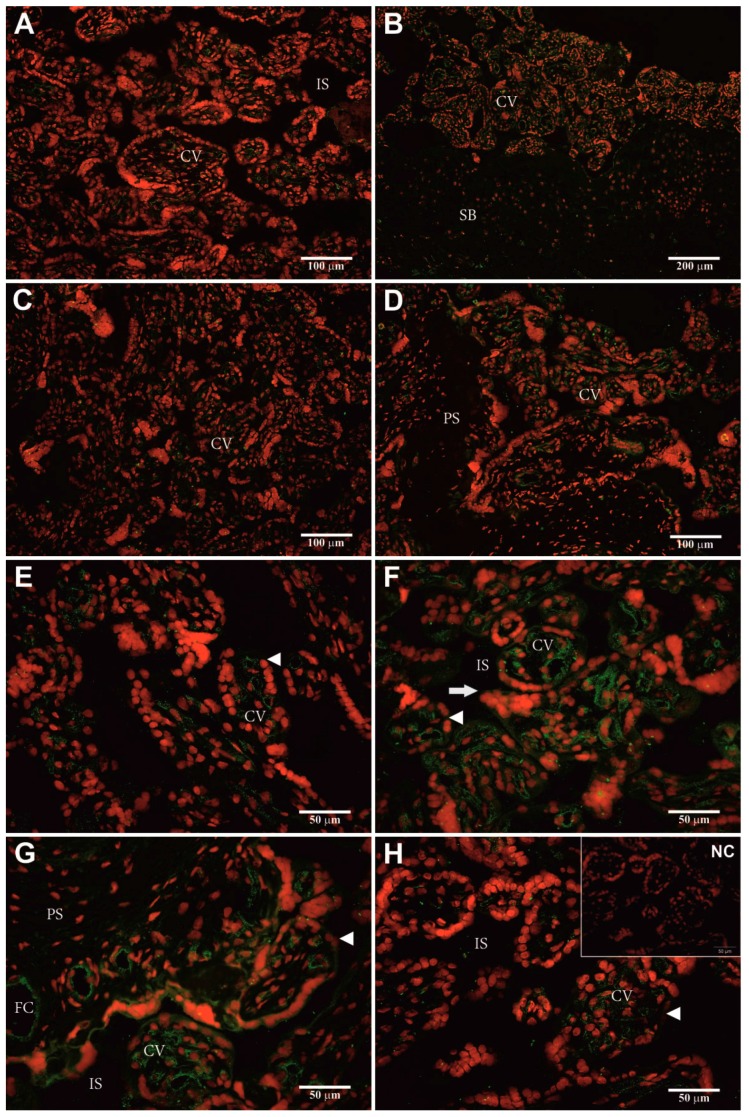
Heterologous immuno-localization of the hPAG-L/pep proteins within sections of human term placenta identified with recombinant anti-porcine PAG (anti-Rec pPAG2) polyclonals (**A–H**), visualized by goat anti-rabbit IgG-conjugated with Alexa 488 fluorophore (green) among all nuclei stained by propidium iodide (red). Human placenta section—used as a negative control (NC; insert in **H**) with omitted polyvalent anti-pPAG-P polyclonals. The size bars are 50 µm (**E**–**H** and NC), 100 µm (**A**,**C**,**D**) and 200 µm (**B**). Abbreviations: arrowheads—syncytiotrophoblast; arrows—clusters of the syncytiotrophoblast nuclei; IS—intervillous space; CV—chorionic villi; SB—stratum basale; PS—placental septa; FC—fetal capillary; NC—negative control.

Heterologous dF-IHC with anti-pPAG-P (Figure 4A–H) and anti-Rec pPAG2 polyclonals (Figure 5A–H) allowed localization of the hPAG-L/pep proteins within term placental cells. Generally, stronger immuno-positive signals of the hPAG-L/pep (green) were identified with anti-Rec pPAG2 than anti-pPAG-P polyclonals. The strongest immune-positive hPAG-L/pep signals were observed within the analyzed regions of chorionic villi (CV) and villous core (VC), especially within the syncytiotrophoblast nuclei (red) covering the surface of the terminal villous tree (arrowheads in Figure 4A–H and Figure 5E–H). The hPAG-L/pep signals were also detectable (Figure 4E and Figure 5G) within VC surrounding fetal capillaries (FC) close to the intervillous space (IS). No hPAG-L/pep signals were immuno-detected within placental septa (PS), which were infiltrated by decidual cells (Figure 5D) or within the maternally-originated stratum basale (SB; Figure 5B). A negative control did not generate any signal (Figure 4H and Figure 5H).

## 3. Discussion

This study presents pioneering data concerning identification of the human placental *PAG-L/pep* cDNA (Acc. No. KX856064) and protein (60 kDa). In addition, the exonic-intronic structure of entire *hPAG-L/pep* gene has been identified (Acc. No. KX533473). Direct comparison of our data is impossible because similar data are not available. Therefore, our data can only be compared to animal species in which the PAGs have already been identified.

### 3.1. Identification of hPAG-L Transcript

The novel 1364 bp *hPAG-L/pep* cDNA, identified with term placental mRNA, allowed identification of nucleotide homology (at least 56.5%) with other AP members. Previously, nucleotide sequences of the *PAG* cDNAs have only been identified in cattle, sheep, pig, goat, horse, zebra, white-tailed deer, water buffalo, American bison, wapiti, giraffe and the Eurasian beaver [1]. Such a limited number of cloned cDNAs resulted from difficulties during the wild eutherian placenta harvesting required for high-quality RNA and cDNA library. The numbers of the *PAG-L* cDNAs vary between species and are multiple in cattle, sheep, goats and pigs, while a single *PAG-L* cDNA has been identified in the horse, zebra, mouse, cat and beaver [1,2,20]. A possible explanation of this fact might be various placenta types, different requirements for the development of the fetus and special environmental needs in different species.

The identified cDNA allowed an encoded 388 aa hPAG-L/pep polypeptide precursor (Figure 1; Table 1, Table 2 and Table 3) to be characterized, which contains 15 aa-signal peptide, 47 aa-blocking peptide and 326 aa-mature polypeptide, making it similar to other PAG precursors. Among the identified PAGs, the length of the 15 aa-signal peptides, as well as the 33–38 aa-blocking pro-pieces are very conservative in various species [21,32,33,34]. Different PAG precursors [1] vary in their entire length (375–389 aa), molecular mass (30–90 kDa) and electrostatic properties (4.0–9.08 pI). Our in silico analyses of the hPAG-L/pep precursor (41.977 kDa; Ip = 3.93 pH) also contributed to the enlargement of the diversity and confirmed membership in the PAG family. The identified hPAG-L/pep precursor was also similar to peps that are composed of 15–16 aa signal peptides, 42–46 aa activation segments and 321–332 aa of mature proteins [5,35].

We are aware that the identified placental *hPAG-L/pep* transcript is identical to another human AP (*pep A*); thus, our *hPAG-L* should be classified as an catalytic active form. Presently, we can expect that the *hPAG-L/pep* and *pep A* are similarly activated by degradation of placental or gastric polypeptide precursors due to an identical blocking peptide sequence (Table 2). Such expectation may confirm equal/similar aa homology (56.4–67.6%) of *hPAG-L/pep* with *peps C* and *F* as well as other catalytically active *PAGs* (*fPAG*, *pPAG2*, *CfPAG*, *ePAG* and *bPAG2*).

High *N*-glycodiversity is very common in the PAGs [1], but in pepsinogens it occurs occasionally and no more than two N-glycosylation sites are generally present [36,37]. Surprisingly, within the aa sequence of the hPAG-L/pep precursor, no potential sites of *N*-glycosylation were predicted. Thus, it confirms that the hPAG-L/pep precursor is different from pep A.

Due to the conserved sequences of two aspartic acids (D) located within two domains (NH_2_- and COOH-terminal), creating the substrate binding cleft, the hPAG-L/pep precursor was classified as a catalytically active AP member, similar to human pep A (Figure 1; Table 3). The PAG-Ls identified in the mouse [38], horse, zebra, cat [21,39] and beaver are also classified as active APs [20]. Within the diversified PAG family in species with multiple PAG members, either potentially active as well as potentially inactive forms exist [1,2,32,40,41]. Multiple aa substitutions within both domains contribute to a loss of catalytic activity of many PAGs [1,3,33,42].

Presumably, some similarities of the PAG-L family in the human and some Rodentia species (beaver or mouse) resembled discoid placenta type and potentially comparable requirements of developing fetuses in those taxa.

Most *PAG/PAG-L* cDNAs share relatively a higher nucleotide homology with each other than to pepsinogens [2]. Interestingly, the *hPAG-L/pep* shares higher homology with pepsinogens than other *PAGs*, similar to *CfPAG-L* and *ePAG* [20,21]. Pepsins were initially considered to be restricted to the stomach of many vertebrates [5]; however, in lower vertebrates, progastricsin (also known as pep C) was also found in the esophageal mucosa of the frog [43] as well as larval pepsinogen cDNA in whole bodies of the pufferfish [44]. Phylogenetically, *peps F* and *PAGs* belong to the same cluster [2,5]. The high (99%) homology of the identified *hPAG-L/pep* cDNA to the human *pep A* indicates that both genes are very similar but are two related *AP* genes with completely different expression.

### 3.2. Identification of hPAG-L Exonic-Intronic Structure

Because data concerning *PAGs* in human genome are not available, the presently obtained results can only be compared to studies performed in some animal species. The entire identified *hPAG-L/pep* gene sequence (9330 bp; Acc. No. KX533473) comprises a structure of nine exons and eight introns (Figure 2). The location of the two D residues, within exons 3 and 7 of *hPAG-L/pep*, is similar to other *PAGs*, which is specific for the catalytic cleft of all APs. To date, the entire structure of the *PAG* genes has only been identified in three species (cattle, pig and beaver). The length of the *hPAG-L/pep* exons (1–9) is greatly similar to exon lengths of *bPAG1*, *bPAG2*, *pPAG2* and *CfPAG* (Table 4) or even the same (especially exons: 3, 4, 8), whereas other exons vary. The gDNA alignments of the *hPAG-L/pep* exons with *bPAG1*, *pPAG2* and *CfPAG-L* revealed high homology in the range of 52.1–78.6% (Table 6). However, the length of the *hPAG-L/pep* introns (A–H) varied from previously discovered *PAGs*, except in the length of intron G for *hPAG/pep* and *bPAG1* or *bPAG2*, as well as their total lengths (Table 4). Furthermore, the pairwise sequence alignment of intronic regions in the aforementioned *PAGs* revealed generally lower homology (25.4–58.5%; Table 6).

Previously, Southern hybridization of gDNA (with selected restrictases) revealed a diversified number of the *PAG-L* genes in some eutherian species, e.g., the elk, yak, wildebeest, impala, several other antelopes [33], the pig, goat, horse, cow, sheep, deer and wild boar and bisons [1]. Southern blot of amplicons also revealed the PAG-L family in the alpaca, the dromedary and the Bactrian [45]. Sequence identification and comparison of cDNA and gDNA enabled defining the exonic-intronic boundaries for only four *PAGs*. So far, multiple bovine *PAG* cDNAs [33,42] have allowed the identification of the *bPAG1* gene (8095 bp) as the first representative with an identified exon-intron structure, with an intron length ranging from 87 bp to 1.8 kbp [17]. Identification of the *pPAG1* and *pPAG2* cDNAs [32] has also led to identification of the *pPAG2* gene structure [19]. The *pPAG2* belongs to the *pPAG2-L* subfamily together with other members: *pPAG4*, *6*, *8*, *10* and they constitute catalytically active APs. However, potentially inactive members of the *pPAG1-L* subfamily, *pPAG3* and *5*, have also been identified [40]. The *pPAG2* structure [19] encompasses nine exons (99–200 bp) and eight introns (A–H; 85–1.8 kbp). The entire length of the *pPAG2* with a promoter region is equal to 8755 bp [19,46]. Recently, *CfPAG-L* (Acc. No. KX377932) was discovered in the Eurasian beaver (7657 bp) as an AP member containing nine exons and eight introns [20]. The lengths of the *hPAG-L/pep* (56–200 bp), as well as *CfPAG-L* (59–200 bp) exons, are similar to the other known *bPAG1*, *bPAG2* and *pPAG2*, although the length of the introns differ from previously identified *PAG*.

Since the results obtained in this study are consistent with the exon-intron structures (length and homology alignment) of four previously described *PAGs* and other *APs*, the identified *hPAG-L/pep* was assuredly classified as a new AP member. However, the high homology of the *hPAG-L/pep* to the pep A family in various species is also a novel finding. The multigenic AP family is widely distributed in various taxa and emerged from duplication or fusion of the paralogous progene [5,6]. In mammals, the major AP members are well-known *pepsinogen* genes classified as *A*, *B*, *C* and fetal forms known as *pepsinogen F* [35,47]. Complete gene structures have been determined, e.g., for human *pep A* [48], *C* [49] and *prochymosin* [50]. The structure is conserved among *APs*, including *PAGs*, *pepsinogens*, *cathepsins D*, *E*, and *renin*, suggesting evolution from a common ancestral gene [51].

### 3.3. Identification of hPAG-L Proteins

Western blotting (Figure 3), with anti-pPAG-P and anti-Rec pPAG2 polyclonals, identified a uniform cellular protein profile of native hPAG-L/pep isoform (60 kDa) in term singleton placentas. Similar data are unfeasible in humans. In animals, multiple heterogeneous secretory PAG isoforms, 43–70 and 45–85 kDa released by placental explants, have been found in the pig and cattle, respectively [34,52]. In the pig, gestation-stage dependent diversity of glycosylated forms of the pPAG proteins occurs, which contain an average of 9.66% of N-linked oligosaccharides [53]. In the bPAG family, oligosaccharide heterogeneity is caused by diversified tetra-antennary glycans [54]. In addition, three purified PAG isoforms (72, 74 and 76 kDa) secreted by the placenta of the American bison have been sequenced [55]. However, in the European bison, among two major groups (43–45 and 67–69 kDa) of immuno-detected secretory PAG isoforms [52], eleven novel diversified pregnancy-stage dependent (45–129 day post coitum–dpc) EbPAGs (50–71 kDa) have been sequenced [56]. Various PAG isoforms also exist in species of the Cervidae order: 33–55 kDa in the white-tailed deer [41], 39–62 kDa in the fallow deer [57], and dominant 55 kDa fraction-specific isoforms for different pregnancy stages (50–200 dpc) in the European moose [58]. It seems that such diversity of multiple PAGs originated from gene duplication during the evolution of different species.

### 3.4. Identification of Cellular hPAG-L Localization

Double-labeling heterologous immuno-detection revealed the strongest positive hPAG-L signals within the chorionic villi, localized especially within the syncytiotrophoblast cells (Figure 4 and Figure 5). Similar data are unachievable in humans. Localization of the hPAG-L expression may be directly compared only with results for the beaver (a discoid-placenta type species) in which CfPAG-L signals are found either in regular or giant trophectodermal cells [59].

In other animals, cellular expression of the PAGs was previously mostly localized in embryo-originated chorionic cells in some species of the Artiodactyla order, with cotyledonary (bovine, bison, white-tailed deer, moose) or the diffuse (porcine, alpaca, camels) placenta types, as described below. In the pig, pPAG expression is restricted to diversified chorionic cell layers throughout (16–61 dpc) placenta development [60]. In ruminants, multi-nucleated, enlarged and multi-granulated trophectodermal cells expressing PAGs have been observed in the white-tail deer [1], while in placentomes of the European bison, the EbPAGs were localized in apical regions of cotyledonary villi folds [61]. Similarly, in camelids during alpaca pregnancy (150–347 dpc), *Lama pacos*–LpPAGs are present in the trophectoderm cell layer and within very rare giant cells [62]. In the term placenta of both camels, CdPAG (*Camelus dromedarius*—dromader) and CfPAG (*Camelus ferrus*—Bactrian) are present in the cytoplasm of the outer folded layer of the mononuclear trophectodermal cells, mostly at the apex of the placental folds [63]. In the moose (*Alces alces*), AaPAG-L signals are related to placentome growth (50–200 dpc) and are localized in the trophectodermal cells, especially within secretory granules [58]. Despite the morphological and developmental divergences of various placenta types, the localization of the hPAG-L/pep resembled chorionic expression previously determined in other mammals.

This study describes pioneering identification of novel aspartic proteinase named *hPAG-L/pep* in the genome (Acc. No. KX533473; 9330 bp), placental transcriptome (Acc. No. KX856064; 1364 nt mRNA) and proteome (60 kDa) of the human. The expression of the hPAG-L/pep in chorionic cells can influence the regulation of placental development. The identified placental glycoprotein presumably can be used as a novel biomarker for prenatal pregnancy diagnosis of embryo/fetus well-being by a noninvasive test based on concentration measurement in peripheral maternal blood, similar to β-hCG test in the human as well as various PAG tests in the ruminants. In addition, the identified cDNA (ORF) and 9-exonic and 8-intronic gDNA sequences provide a major pattern of SNPs/InDels required for a novel marker preparation profitable for genotyping and detection of various genomic disorders in embryo/fetus and mother, similar to our report on SNPs/InDels for the pig [46,64] and the European moose [58]

## 4. Materials and Methods

### 4.1. Ethics Statement and Collection of Samples

All clinical samples (placentas and blood) were collected at the Clinical Ward for Gynecology, Obstetrics and Oncological Gynecology at the Regional Specialist Hospital in Olsztyn, following informed written consent from the parturient women. The study protocol was approved by the Bioethics Committee of the Warmia-Mazury Medical Chamber (OIL.164/15/Bioet; 2 April 2015) in Olsztyn, Poland. Only healthy mothers, after uncomplicated single pregnancy and without diagnosed medical conditions were selected for this study. Term placentas (*n* = 2) were collected from the women who underwent scheduled Caesarean section. Whole blood samples from both men (*n* = 3) and women (*n* = 3) were also collected from jugular veins. Placental tissues were immediately preserved in liquid nitrogen and blood samples were placed on ice and transported directly to the laboratory. Samples of blood were centrifuged (3500× *g*) for 30 min at 4 °C, plasma was discarded and the buffy coat of the white cells, as well as placental tissues were stored at −70 °C until further analyses.

### 4.2. Total RNA Extraction

Total RNA was isolated from term placental fragments, using a QiagenRNeasy Kit in conjunction with the QiagenRNase-Free DNase Set (Qiagen, Hilden, Germany), according to the manufacturer’s recommendations. RNA quality was evaluated via microfluidic electrophoresis (2100 Bioanalyzer; Agilent Technologies, Santa Clara, CA, USA). Only a high RNA integrity number (RIN > 8.0) of each sample was accepted for high throughput mRNA sequencing (RNA-seq).

### 4.3. High Throughput mRNA and Bioinformatics

Complementary DNA (cDNA) libraries were constructed using the protocol of TruSeq Stranded mRNA LT Sample Prep Kit (Illumina, San Diego, CA, USA) involving the following steps: RNA purification and fragmentation, synthesis of the first and the second strand of cDNA, 3′ adenylation and adaptor ligation. After amplification and quantification (KAPA Library Quantification Kit, Illumina), the cDNA libraries were indexed, diluted and pooled in equimolar ratios.

The paired-end sequencing was performed on the HiSeq2500 (Illumina). The quality of raw reads (2 × 100 bp reads) was controlled by FASTQC software v.0.11.2 (https://www.bioinformatics.babraham.ac.uk/projects/fastqc/). FLEXBAR software v.2.5 (https://github.com/seqan/flexbar) was used for trimming the Illumina adaptors and poly(A) stretches. All reads shorter than 32 bp and reads with a PHRED quality score lower than 10 were then disqualified from the dataset. The trimmed reads were used for de novo assembly with TRINITY software v.r20140717 (https://github.com/trinityrnaseq/trinityrnaseq/releases). To select potential human PAG-L (hPAG-L) sequences, reconstructed contigs were searched against a database (https://blast.ncbi.nlm.nih.gov/Blast.cgi). The sequencing data from this study have been submitted (http://www.ncbi.nlm.nih.gov/sra) to the NCBI Sequence Read Archive (SRA) under Accession no. BioProject ID: PRJNA326064.

### 4.4. Capillary Sequencing

Capillary sequencing was performed to confirm the coding sequence of the placental *AP*, identified by RNA-Seq. Briefly, total RNA (from the same samples that were used for RNA-Seq) was transcribed to cDNA in two-step RT-PCR using an Enhanced Avian HS RT-PCR Kit (Sigma-Aldrich, USA). The first strand cDNA was synthesized with dNTPs, and random hexamers were used as primers. PCR amplification of target cDNA templates to obtain *hPAG-L* amplicons was performed with specific primers (Table 7) designed by applying Geneious^®^ 8.1.7 software (Biomatters Ltd., Auckland, New Zealand) and Oligo Calc: Oligonucleotide Properties Calculator (http://www.basic.northwestern.edu/biotools/oligocalc.html), basing on *PAG-L* sequence identified using RNA-Seq.

The obtained amplicons of examined *hPAG-L*, parallel to porcine *PAG10* (*pPAG10*) cDNA—used as a positive control and negative control (without templates)—were separated in 1.5% agarose gels, along with a marker (100–3000 bp; Thermo Fisher Scientific, Waltham, MA, USA), UV-visualized using Midori Green Nucleic Acid Staining Solution (NIPPON Genetics Europe GmbH, Dueren, Germany) and archived (G:Box, SynGen, Sacramento, CA, USA). Gel-out purified *hPAG-L* amplicons were used as templates for capillary sequencing (3130 Genetic Analyzer, Applied Biosystems, Foster City, CA, USA) in both sense and antisense directions. Amplicon labeling was performed with the BigDye Terminator v3.1 Cycle Sequencing Kit (Applied Biosystems), under the following conditions: initial denaturation (at 96 °C for 1 min) and 30 cycles of amplification (96 °C/10 s, 50 °C/5 s, 60 °C/4 min). Each labeling mix (20 μL) was composed of 12 μL (5–10 ng) of amplicon template, 1.2 μL Ready Reaction Mix, 4 μL BigDye Terminator v1.1/3.1 Sequencing buffer (5×), 2 μL of each primer and 0.8 μL H_2_O. The labeled hPAG-L amplicons were purified with the BigDye X Terminator Purification Kit (Applied Biosystems) and separated in capillaries filled with POP-7™ polymer (Applied Biosystems). The obtained *hPAG-L* sequences were analyzed by Geneious^®^ 8.1.7. In addition, in silico analyses of the cDNAs were performed applying the following online tools: http://www.cbs.dtu.dk/services/SignalP/; http://prosite.expasy.org; http://www.cbs.dtu.dk/services/NetNGlyc/.

### 4.5. Genomic Identification of the hAP/PAG-L Sequence

Genomic DNA (gDNA) templates (*n* = 6) were isolated from the leukocytes with the use of a commercially available kit (Sherlock AX, A&A Biotechnology, Gdynia, Poland). Only high quality gDNA templates (700 ng) were used for PCR amplifications of the *hPAG-L* gene fragments. In order to identify either initial or partial nucleotide sequence of the *hAP/hPAG-L*, the gDNA amplicons were produced with 19 pairs of specific homologous primers (Table 8), designed on the basis of the *hPAG-L* cDNA sequence originating from the aforementioned RNA-seq. JumpStart™ Taq ReadyMix™ (Sigma-Aldrich, St. Louis, MO, USA) was used for efficient PCR amplification, under the following conditions: initial activation (95 °C/2 min), followed by 40 following cycles: 95 °C/1 min for the denaturation of gDNA templates, 60 °C for primer annealing (1 min) and 72 °C/4.5 min for amplicon synthesis. The obtained *hPAG-L* gDNA amplicons were electrophoresed, gel-out purified, subjected to capillary sequencing and analyzed as described above (see Capillary sequencing). Identified cDNA and gDNA sequences of *hPAGL-L* have been deposited in GenBank (Accession nos: KX856064 and KX533473, respectively

### 4.6. Identification of the Exon-Intron Organization of the hPAG-L

To estimate the length of the introns and exons in the *hPAG-L*, the identified sequences were analyzed by NetGene2 v. 2.4 software (www.cbs.dtu.dk/services/NetGene2/) to predict a structure, based on multiple alignments (Geneious^®^ 8.1.7, www.geneious.com and BLAST, https://blast.ncbi.nlm.nih.gov/Blast.cgi) of the entire *hPAG-L* genomic sequence with the identified cDNA (see above).

### 4.7. Cellular Placental Protein Extraction

Cellular proteins were isolated as previously described for other species [58,65]. Briefly, the frozen human placental tissues (*n* = 2) were homogenized on ice and lysed by alkaline buffer (Total Protein Extraction Kit, Genoplast, Rokocin, Poland). The obtained protein homogenates of each placental sample were concentrated (3000 rpm/4 °C) by ultra-filtration in Centriprep-10 cartridges (>MWCO 10 kDa; Amicon, Billerica, MA, USA) until a 0.5 mL of final volume was received. Total protein concentration was determined by the standard Bradford procedure. Concentrated placental proteins (10 µg/line) were separated by denaturing polyacrylamide electrophoresis (SDS-PAGE, 12.5% gels), parallel to porcine placental proteins (positive control for Western blotting), endometrial proteins of cyclic pigs (negative control) and a molecular marker (10–250 kDa; Fermentas, Waltham, MA, USA). Electrophoresed proteins were stained with Coomassie Brilliant Blue (CBB) to identify total human placental protein profiles.

### 4.8. Western Blotting

Duplicates of PAGE-separated human cellular proteins, porcine secretory chorionic (positive control; 77 dpc) and porcine secretory endometrial proteins (negative control; 10 day of cycle) were transferred onto 0.45 µm nitrocellulose membranes (Optitran BA-S58, Whatman, GE Healtcare Life Science, Issaquah, MA, USA) and then analyzed by Western blotting to identify human placental PAG-L fractions by the heterologous—ht (cross-species) immuno-detection described previously [66,67]. Briefly, blotting was performed with primary rabbit polyvalent anti-pPAG polyclonals, raised against various porcine antigens (anti-pPAG-P; 1:300) and against recombinant pPAG2 antigen (anti-Rec pPAG2; 1:50). Immuno-complexes were identified by secondary mouse anti-rabbit IgG monoclonals–conjugated with alkaline phosphatase (1:100,000). The immuno-complexes were visualized with the use of nitro blue tetrazolium (NBT) and 5-bromo-4-chloro-3-indolyl-phosphate (BCIP) as standard substrates for alkaline phosphatase activity detection. Gels and blots were photographed and archived (GBox, SynGen, Sacramento, CA, USA). Methodological details, as full description and validation methods of applied polyclonals, are provided in Supplementary material.

### 4.9. Heterologous Double Fluorescent Immunohistochemistry (htdF-IHC)

Placental explants were cryo-sectioned (−20 °C; 6 μm), fixed, dehydrated and then subjected to htdF-IHC, as previously described [61,62,63]. Briefly, the htdF-IHC was performed with the aforementioned anti-pPAG-P (1:300) and anti-Rec pPAG2 (1:50) polyclonals. Parallel negative controls were performed without the primary antisera. Double immunostainings were visualized with secondary goat anti-rabbit polyclonals (1:1000)–conjugated with Alexa 488 fluorophore (A488; green) and all nuclei of placental cells were stained with propidium iodide (PI; red).

## 5. Conclusions

Our discerning and comprehensive studies provide novel data identifying the placental *hPAG-L/pep* transcript, gene structure and chorionic protein in humans. Our pioneering data extend the present knowledge of the human genome, placental transcriptome and proteome, which may contribute to establishing a new diagnostic tool for examination of various disturbances during human pregnancy, with growing interest from both scientific and clinical perspectives.

## Figures and Tables

**Table 1:** Signal peptide (SP) sequence homology of the hPAG-L/pep polypeptide precursor to various aspartic proteinases.

Gene Name ^a^	SP Sequence (aa) ^b^	Identity (%)	Positive aa (%)
*hPAG-L/pep*	MKWLLLLGLVALSEC	this study	this study
*hPepsinogen A*	...............	100	100
*bPAG2*	....V..........	93.3	100
*pPAG1*	....VI.........	86.7	100
*mPepsinogen F*	....WV.........	86.7	93.3
*fPAG*	....WV.........	86.7	93.3
*pPAG2*	....VI.......D.	80	100
*ePAG*	...FGV....T....	73.3	80
*CfPAG-L*	...IVVA.LC.P.L.A	37.5	62.5
*hCathepsin E*	..T....L..L.ELGEAQG	60	60
*hPepsinogen C*	...MVVV-..C.QLLEA	40	66.7
*hNapsin A*	QPL....P.LNVEPSGA	33.3	46.7
*hRenin*	PR.G..--.LLWGS.TFG	33.3	46.7
*hCathepsin D*	.QPSS..P.ALCLLAAPASA	26.7	33.3

^a^ aa—Amino acids, b—bovine, e—equine, f—feline, h—human, m—mouse, p—porcine, Cf—beaver; ^b^ Identical aa are dotted. Gaps (–) have been inserted to provide maximal alignments.

**Table 2:** Blocking peptide aa sequence homology of the human PAG-L/pep polypeptide precursor to various aspartic proteinases.

Gene Name ^a^	Blocking Peptide Sequence (aa) ^b^	Identity (%)	Positive aa (%)
*hPAG-L/pep*	IMYKVPLIRKKSLRRTLSERGLLKDFLKKHNLNPARKYFPQWEAPTL	this study	this study
*hPepsinogen A*	...............................................	100	100
*hPepsinogen C*	AVV....KKF..I.E.MK.K...GE..RT.KYD..W..R.GDL	46.5	65.1
*fPAG*	-LVTI..T.V..M.EN.R.KDR.....EN.PY.L.Y.FVD	43.6	59
*pPAG2*	-LVMI..TKV..V.ES.R.K....N...E.PY.MIQNL	43.2	67.6
*CfPAG-L*	AISRI..RKA..V.Q..K.K...EE...T.KYD..Q..LANNFGDF	41.3	65.2
*pPAG1*	-LVII..TKV..I.EN.R.KD..LN...E.PY.MIQ.F	40.5	64.9
*ePAG*	-LVTI..VKI....EN.R.KDM..EY.E.YPFRL	36.4	66.7
*mPepsinogen F*	-LV.I..MKI..M.EN.R.SQV...Y.E.YPRSR.HVLLE.RRN.	36.4	59.1
*bPAG2*	.VIL-..KKM.T..E..R.KN..NN..EEQAYRLSKNDS	33.3	56.4

^a^ aa—Amino acids, b—bovine, e—equine, f—feline, h—human, m—mouse, o—ovine, p—porcine, Cf—beaver; ^b^ Identical aa are dotted. Gaps (–) have been inserted to provide maximal alignments.

**Table 3:** Comparison of the aa sequence of NH_2_- and COOH-terminal domains in human PAG-L/pep polypeptide precursor to various aspartic proteinases.

Gene Name ^a^	NH_2_-Domain ^b^	Identity (%)	COOH-Domain ^b^	Identity (%)
*hPAG-L/pep.*	VVFDTGSSNLWV	this study	AIVDTGTSLLTG	this study
*hPepsinogen A*	.............	100	............	100
*hCathepsin D*	............	100	.........MV.	83.3
*hPepsinogen C*	.L..........	91.7	...........V	91.7
*hCathepsin E*	.I..........	91.7	.........I..	91.7
*CfPAG-L*	.L..........	91.7	G..........V	83.3
*TrNothepsin*	........D...	91.7	.........IA.	83.3
*hNapsin A*	.A..........	91.7	..L......I..	83.3
*pPAG2*, *4*, *6*, *10*	........D...	91.7	........MLH.	75
*oPAG2*	........D...	91.7	.L.......IH.	75
*bPAG2*	.......A....	91.7	.LL.....MIY.	58.3
*hRenin A*	.........V..	91.7	.L....A.YIS.	58.3
*mPepsinogen F*	..L.....V...	83.3	G.M.........	83.3
*fPAG*	.I......D...	83.3	..I.......I.	83.3
*ePAG*	.I.....AD...	75	..........L.	91.7
*zPAG*	.I.....AD...	75	..........L.	91.7
*pPAG1*, *3*, *5*	.I...A..D...	75	..L.S.SAF.L.	50

^a^ aa—Amino acids, b—bovine, e—equine, f—feline, h—human, m—mouse, o—ovine, p—porcine, z—zebra, Tr—pufferfish, Cf—beaver; ^b^ Identical aa are dotted and aspartic acid (D) located within domain creating the substrate binding cleft is underlined.

**Table 4:** Exonic and intronic length of the *hPAG-L/pep* compared to *bPAG1*, *bPAG2*, *pPAG2* and *CfPAG-L* genes.

Sequence Length (bp)					
Gene Segment	*hPAG-L/pep*	*bPAG1*	*bPAG2*	*pPAG2*	*CfPAG-L*
Exon 1	56	53	53	53	59
Intron A	486	1100	1300	1093	1937
Exon 2	163	151	151	166	160
Intron B	2233	1000	1000	1324	385
Exon 3	118	118	118	118	118
Intron C	869	100	100	90	917
Exon 4	119	119	119	119	119
Intron D	675	1200	1200	1124	451
Exon 5	200	194	194	200	200
Intron E	1351	900	1100	927	1138
Exon 6	117	117	117	117	123
Intron F	1131	1900	1700	1455	288
Exon 7	145	142	142	136	148
Intron G	102	100	100	85	681
Exon 8	99	99	99	99	99
Intron H	1119	1700	1700	292	603
Exon 9	150	150	150	156	147
Total length	9133	9143	9343	8031	7573

**Table 5:** Characteristics of exon-intron junctions within the *hPAG-L/pep* gene.

Donor Splice Sites					Acceptor Splice Sites				
Exon	5′→3′	Phase	Intron	5′→3′	Intron	5′→3′	Phase	Exon	5′→3′
1	TCATGTACAA	0	A	GTGAGTCCGG	A	CAAACCACAG	2	2	GGTCCCCCTC
2	CTACCTGGAT	0	B	GTGAGTGTGC	B	GCCTGGACAG	0	3	ATGGAGTACT
3	CTTGCCTGCA	1	C	GTAAGTGCCC	C	GTCCTTGCAG	1	4	CCAACCACAA
4	CACTGTCCAG	0	D	GTGGGCACCT	D	CCCCACCCAG	0	5	GTTGGAGGCA
5	ACCTCAGCGC	2	E	GTAAGTTGAG	E	CTTTCCACAG	2	6	CGATGACCAG
6	CCGTGGACAG	2	F	GTGAGACTGC	F	TTGCCCTCAG	2	7	CATCACCATG
7	AGATGGCGAC	0	G	GTGAGTCCAG	G	CTCTTTCCAG	0	8	ATGGTGGTCA
8	CATCCTGCAG	0	H	GTGAGGAGGC	H	TTTTCTCCAG	0	9	AGCGAGGGGA

**Table 6:** Homology of the *hPAG-L/pep* exons and introns with *bPAG1*, *pPAG2* and *CfPAG-L* genes.

Pairwise Identity (%)			
*hPAG-L/pep*	*bPAG1*	*pPAG2*	*CfPAG-L*
Exon 1	78.6	75.5	63.3
Intron A	49.8	51.1	50.3
Exon 2	52.1	58.8	59.6
Intron B	51.7	50.7	52.7
Exon 3	69.5	74.6	71.2
Intron C	55.6	54.5	25.4
Exon 4	59.7	58.4	64.7
Intron D	52.5	52.2	52.7
Exon 5	58.5	65.3	65.5
Intron E	51.3	53.3	50.9
Exon 6	61.9	69.7	56.9
Intron F	51.2	51.0	50.3
Exon 7	62.0	63.3	64.7
Intron G	58.5	54.2	54.7
Exon 8	67.7	66.7	70.3
Intron H	52.9	52.3	51.8
Exon 9	58.7	61.4	61.5

**Table 7:** Specific primers applied for the amplification of human *PAG-L* cDNA.

Primers Name		Sequence (5′–3′)	Position (bp) ^a^	Amplicon Length (bp) ^a^
1	MMstart	AGTTGGGACCCGGGAAGA	1–18	1363
	MMutrR	TCCACAAAACCTGTTTCAGTG	1343–1364	
2	MM2s	TCATCAGAAAGAAGTCCTTGAG	85–106	496
	MM5as	TAGGCCAGSCCCAKGATGCCATC	558–580	
3	MM3s	GCTCCTCCAACCTGTGGGT	307–325	560
	MM7as	CAGAGAGGTGCCKGTGTCMACAA	844–866	
4	MM5s	GATGGCATCMTGGGSCTGGCCTA	558–580	564
	MM9as	GAAGACATCWCCMAGGATCCAA	1100–1121	
5	MM7s	TTGTKGACACMGGCACCTCTCTG	844–866	520
	MMutrR	TCCACAAAACCTGTTTCAGTG	1343–1364	

^a^ Position and amplicon length was estimated according to the human *PAG-L* cDNA sequence identified using RNA-seq.

**Table 8:** Specific primers applied for identification of the human *PAG-L* genomic sequence.

Primers Name		Sequence (5′–3′)	Position (bp) ^a^	Amplicon Length (bp) ^a^
1	MMstart	AGTTGGGACCCGGGAAGA	1–18	725
	MM2as	ATCCAGGTAGTTCTCCAGGG	706–725	
2	MM2s	TCATCAGAAAGAAGTCCTTGAG	571–592	1789
	MMintronBr	ATTCTCCTGCCTCAACCTCCCAA	2337–2359	
3	MMintronB	CTCCGCATAGCCTGATCCCTT	1180–1200	1180
	MMintronBr	ATTCTCCTGCCTCAACCTCCCAA	2337–2359	
4	MMintronB3	CCTCCTGCAGATATTGTATGTCC	1429–1451	1616
	MM3as	ACCCACAGGTTGGAGGAGCC	3025–3044	
5	MMintronB2	TGTGAGGAATGAAGGAAAAGATGG	2840–2863	1533
	MMintronDr	GGTGCTGCATGTCGGGAGAA	4353–4372	
6	MMintronC	GCTGTAGAATAGCCCACCAGG	3381–3401	992
	MMintronDr	GGTGCTGCATGTCGGGAGAA	4353–4372	
7	MMintronC	GCTGTAGAATAGCCCACCAGG	3381–3401	1663
	MMintronEr	AAGACCCTCTCCATCGCACCCA	5022–5043	
8	MMintronD(N)	AGTCCTGCATGAGATGAACCA	4636–4656	1284
	MMintronEr3	CTTAAGGACTTGAGGGTGGAGGTC	5896–5919	
9	MMintronE3	GCACAACTCAAATGTCATCAGCCA	5178–5201	742
	MMintronEr3	CTTAAGGACTTGAGGGTGGAGGTC	5896–5919	
10	MMintronE3	GCACAACTCAAATGTCATCAGCCA	5178–5201	1325
	MMintronFr2	CTGGGGGGATTCTGGAAAGCTGA	6480–6502	
14	MM6sens	AGTGGCAGCGTGGTGATCTTTG	6301–6322	1311
	MM7as	CAGAGAGGTGCCKGTGTCMACAA	7589–7611	
15	MMintronF2	TCAGCTTTCCAGAATCCCCCCAG	6480–6502	1132
	MM7as	CAGAGAGGTGCCKGTGTCMACAA	7589–7611	
16	MMintronF	TGGATGGGTGGGGAAGAAATGT	7438–7459	1650
	MM9as	GAAGACATCWCCMAGGATCCAA	9066–9087	
17	MM8s	GACATCGTCTTCACCATCAAT	7822–7842	1266
	MM9as	GAAGACATCWCCMAGGATCCAA	9066–9087	
18	MMintronF	TGGATGGGTGGGGAAGAAATGT	7438–7459	405
	MM8as	ATTGATGGTGAAGACGATGTC	7822–7842	
19	MM992	GAGCTGCATCAGTGGCTTCC	9012–9031	318
	MMutrR	TCCACAAAACCTGTTTCAGTG	9309–9329	

^a^ Position and amplicon length was estimated according to the human *PAG-L* gDNA identified using capillary sequencing.

## Supplementary Materials

Supplementary materials can be found at www.mdpi.com/1422-0067/18/6/1227/s1.

## Abbreviations

PAG	Pregnancy-associated glycoprotein family
PAG-L	PAG-like
hPAG-L	Human PAG-L
Pep	Pepsinogen
RNA-seq	RNA sequencing
aa	Amino acids
D	Asparagine (Asp)
cDNA	Complementary DNA
gDNA	Genomic DNA
bPAG	Bovine PAG
pPAG	Porcine PAG
CfPAG-L	Beaver PAG-L
EbPAG	European bison PAG
LpPAG	*Lama pacos* PAG
CdPAG	*Camelus dromedarius* PAG
CfPAG	*Camelus ferrus* PAG
AaPAG-L	*Alces alces* PAG
RIA	Radioimmunological test
ELISA	Immunoenzymatic test
RT-PCR	Reverse transcriptase PCR
dNTP	Deoxynucleotide
MWCO	Molecular weight cut-off
SDS-PAGE	Denaturing polyacrylamide electrophoresis
CBB	Coomassie Brilliant Blue
NBT	Nitro blue tetrazolium
BCIP	5-Bromo-4-chloro-3-indolyl-phosphate
ht	Heterologous (cross-species)
anti-pPAG-P	Polyclonals raised against various porcine antigens
anti-Rec pPAG2	Polyclonals raised against recombinant pPAG2 antigen
htdF-IHC	Heterologous double fluorescent immunohistochemistry
A488	Alexa 488 fluorophore
PI	Propidium iodide
HQ	High quality
CDS	Coding sequence
UTR	Untranslated region
QC	Query Cover
SP	Signal peptide
ID	Sequence identity
CV	Chorionic villi
VC	Villous core
FC	Fetal capillaries
IS	Intervillous space
PS	Placental septa
SB	Stratum basale
NC	Negative control
dpc	Day post coitum
