# Supplementary material for: Identification of Novel Placentally Expressed Aspartic Proteinase in Humans
Source: Int J Mol Sci. 2017 Jun 8;18(6):1227. doi: 10.3390/ijms18061227 (PMC5486050; doi:10.3390/ijms18061227)
Supplement: Supplementary file 1 [file ijms-18-01227-s001.pdf]

Supplementary Materials

The purpose of our study was to investigate whether or not PAG-Like proteins are expressed in human placenta and anti-porcine PAG polyclonals were successfully used for PAG identification in various species (Majewska et al. 2005, 2006, 2008, 2010, 2013; Lipka et al. 2016), thus we expected this approach may be effective also for the human. Antisera applied in our study were previously described and validated (Szafranska et al., 2003; Majewska et al. 2006).

Briefly, the secretory chorionic proteins of pregnant gilts were recovered by precipitation with 20%, 40% and 75% saturation of $(NH_4)_2SO_4$, then the proteins were fractionated and concentrated in Amicon cartridges (MWCO 30 kDa). The separated porcine proteins were analyzed by SDS-PAGE and monitored by Western blotting with well-defined various PAG sera. Selected fractions of proteins obtained from pigs on Days 17–77 of pregnancy and recombinant pPAG2 proteins (prepared on a basis of cDNA and produced in the bacterial system) – have been used as immunogens to immunize crossbred rabbits. Blood samples were collected from rabbits, then crude antisera were initially tested for their titer and bounding of selected antigens. Unspecific binding of each antiserum has been removed through several adsorption with endometrial tissues or proteins produced *in vitro* of non-pregnant pigs, to reduce non-specific binding. In details, non-specific Igs were adsorbed with sliced endometrial tissues of luteal phase gilts, or were adsorbed by endometrial proteins obtained from the culture media. All of the non-specific immunocomplexes for gestation (non-specific Ig with endometrial proteins of luteal-phase cyclic pigs) were removed from purified antisera. The adsorption of each antiserum was performed several times, until non-specific Ig (binding of endometrial proteins of non-pregnant animals) had been removed. Finally, the specificity of each antisera against recombinant pPAG2 or polyvalent antisera against multiple native antigens were examined by Western dot-blottings. As a positive controls we used porcine chorionic proteins produced *in vitro*, BSA, endometrial proteins produced *in vitro* and ovalbumin as a negative controls. Adsorption was completed when the antisera did not show any cross-reactions with endometrial proteins (non-specific for gestation) and then such purified antisera were subjected to immunoblotting of chorionic proteins separated by PAGE analysis or F-IHC analysis (Szafranska et al., 2003). Moreover, specificity of purified polyvalent anti-pPAG sera was confirmed by immunoscreening of various pPAG cDNA clones isolated from a porcine trophoblastic cDNA library (Szafranska et al., 2003). Thus, applied antisera were fully described and validated.
